# Rapid Microwave-Assisted Synthesis of N/TiO_2_/rGO Nanoparticles for the Photocatalytic Degradation of Pharmaceuticals

**DOI:** 10.3390/nano12223975

**Published:** 2022-11-11

**Authors:** Camilo Sanchez Tobon, Ivana Panžić, Arijeta Bafti, Gordana Matijašić, Davor Ljubas, Lidija Ćurković

**Affiliations:** 1 Faculty of Mechanical Engineering and Naval Architecture, University of Zagreb, Ivana Lučića 5, 10000 Zagreb, Croatia; 2 Faculty of Chemical Engineering and Technology, University of Zagreb, Marulićev trg 20, 10000 Zagreb, Croatia

**Keywords:** microwave-assisted method, photocatalysis, N/TiO_2_/rGO, organic micropollutants, UVA light, simulated solar light, visible light

## Abstract

Nanocomposites comprising nitrogen-doped TiO_2_ and reduced graphene oxide (N/TiO_2_/rGO), with different rGO loading qualities, were prepared by a cost-effective microwave-assisted synthesis method. The synthesized materials were broadly characterized by Raman spectroscopy, X-ray diffraction (XRD), infrared spectroscopy (FTIR), photoelectron spectroscopy (XPS), diffuse reflectance spectroscopy (DRS), electron microscopy (SEM-EDS), and nitrogen adsorption/desorption isotherms. Anatase was the only crystalline phase observed for all synthesized materials. The rGO loading did not affect the morphological properties, but it positively influenced the photocatalytic activity of the nanocomposite materials, especially at low rGO loading. Photocatalysts were evaluated via the degradation of specific organic micropollutant (OMP) pharmaceuticals: ciprofloxacin (CIP), diclofenac (DCF), and salicylic acid (SA), under different radiation sources: ultraviolet A (UVA), solar light simulator (SLS), blue visible light (BVL) and cold visible light (CVL). CIP and SA were removed effectively via the synergy of adsorption and photocatalysis, while DCF degradation was achieved solely by photocatalysis. After implementing scavenger agents, photocatalytic degradation processes mainly depended on the specific pollutant type, while irradiation sources barely defined the photocatalytic mechanism. On the other hand, changes in irradiation intensity significantly influenced the photolysis process, while photocatalysis was slightly affected, indicating that irradiation spectra are more relevant than intensity.

## 1. Introduction

Every year, thousands of new chemicals are produced for different applications. At the same time, those chemicals are released into the environment mostly without knowing their possible negative effects on ecosystems and human beings [1]. Among all kinds of pollutants discharged into the water and air without any regulation, organic micropollutants (OMPs) have been of particular interest in the last 20 years due to their possible negative impact on the environment. These OMPs are mainly pharmaceuticals, personal care products, disinfection by-products, endocrine disrupters, and all their transformation by-products [2]. Apart from the fact that these OMPs are not easily removed by conventional wastewater treatment plants and subsequently enter water bodies, some are persistent and can bioaccumulate, being harmful to the aquatic system and representing a threat to public health [3,4,5]. On the other hand, water reuse is becoming a feasible solution to overcome water scarcity and the continuous increment of water stress in many regions worldwide. Therefore, to ensure the safety of water reuse, the complete removal of OMPs must be guaranteed, which could be achieved by coupling new treatment technologies with existing wastewater treatment plants (WWTPs) [6].

Advanced oxidation processes (AOPs) such as ozonation, fenton, photocatalysis, photoelectrocatalysis, sonochemistry, etc., have been considered as excellent alternatives for removing OMPs from wastewater with high oxidation efficiency and no secondary pollution effects [7,8,9,10,11,12]. Among different semiconductors used in photocatalysis, TiO_2_ is actively studied because of its outstanding photocatalytic activity, low cost, excellent chemical stability, and non-toxicity [13,14]. TiO_2_ is activated by light energy, producing reactive oxygen species (ROS), which subsequently can oxidize OMPs non-selectively. However, due to its high energy bandgap (3.2 eV), photoactivation of TiO_2_ occurs only through UV light exposure, hindering its potential under solar radiation (4% UV, 48% Visible). The fast recombination of photogenerated electron/hole pairs that produce ROS can also reduce TiO_2_ photocatalytic activity [15,16]. Therefore, TiO_2_ has been doped with different elements to reduce the recombination rate and/or shift the optical response of TiO_2_ to the visible light range to make TiO_2_ more efficient under solar radiation exposure. Non-metal doping is one of the most feasible options because it is inexpensive and has less leaching toxicity than metal doping. One of the most used and widely studied non-metal elements for TiO_2_ doping is nitrogen [17]. This element can create new energetic levels in TiO_2_ that shift its photoactive response under visible light [18,19]. On the other hand, reduced graphene oxide (rGO) has attracted significant attention as a material for producing TiO_2_ nanocomposites due to its high surface area and good thermal and electronic conductivity that could improve the adsorption and photoactivity of TiO_2_ [20,21]. Recent studies have shown that coupling rGO with TiO_2_ can improve photocatalytic activity, because rGO acts as a sink for electrons, facilitating the separation of photogenerated charges and reducing the recombination effect [22,23,24]. So, integrating nitrogen and rGO into TiO_2_ could be an interesting approach for tackling the two main drawbacks of TiO_2_ mentioned before.

In addition to the doping strategy, the TiO_2_ synthesis method plays an important role due to its influence on structural, morphological, and optical properties and its photocatalytic activity [17,25]. The microwave-assisted method has become an attractive alternative recently because of its shorter synthesis time and lower energy consumption than conventional synthesis methods [26]. Moreover, the microwave-assisted method reduces the gradient temperature inside the reaction vessel, producing a material with more homogeneous properties [27,28].

Therefore, as a continuation of the research reported in [19], this study focuses on the synthesis of N/TiO_2_/rGO photocatalyst via the microwave-assisted method, evaluating the role of rGO content on the morphological properties and testing the photocatalytic activities under different radiation sources. This use of very different irradiation spectra, with very carefully defined spectra intervals, as light sources for photocatalytic experiments and their results comparison should contribute new knowledge to the already known facts about the behavior of the photocatalytic hybrid nanoparticles. The use of different irradiation spectra is necessary, since the relatively low availability of data concerning the examination of hybrid photocatalysts with rGO was noticed. Scavenging species were used to determine the photocatalytic mechanism under different irradiation sources. Additional studies were performed using diverse irradiation intensity data to investigate the feasibility of using natural solar light.

The initial intention of authors was to make an improvement in the sector of wastewater treatment, since it is clear that relevant substances, such as pharmaceuticals, can pass the usual treatment processes at the wastewater treatment facilities. The improvement of the pharmaceutical degradation in wastewaters is both a market and environmental demand. As the model substances, aqueous solutions of ciprofloxacin (CIP), diclofenac (DCF), and salicylic acid (SA) were used. The DCF and CIP were included in the first and second EU watchlist of substances for union-wide monitoring in the field of water policy, respectively [29,30]. Meanwhile, SA is a precursor and a transformation by-product of acetylsalicylic acid, one of the most widely used analgesics and additives for several healthcare products [9]. The combination of all these results could provide the readers and scientific community with interesting and useful information, as well as challenges and future perspectives in TiO_2_ photocatalysis for use in the real wastewater treatment sector.

## 2. Materials and Methods

### 2.1. Materials

Titanium (IV) isopropoxide (TTIP, 97%, Sigma-Aldrich, St. Louis, MO, USA), acetylacetone (AcAc, ≥99%, Honeywell, Charlotte, NC, USA), absolute ethanol (EtOH, p.a., Grammol, Zagreb, Croatia), urea (99.5%, Sigma-Aldrich, St. Louis, MO, USA), graphene oxide water dispersion (0.4 wt.% concentration, Graphenea, Cambridge, MA, USA), ascorbic acid (p.a., Sigma-Aldrich, St. Louis, MO, USA), ciprofloxacin (CIP, 98%, Acros Organics, Waltham, MA, USA), diclofenac sodium salt (DCF, Sigma-Aldrich, St. Louis, MO, USA), salicylic acid (SA, p.a., Grammol, Zagreb, Croatia), *p*-Benzoquinone (≥99%, Sigma-Aldrich, St. Louis, MO, USA), formic acid (≥95%, Sigma-Aldrich, St. Louis, MO, USA) and methanol (≥99.9%, Sigma-Aldrich, St. Louis, MO, USA) were used as received without further purification. Deionized water of ultrapure quality (electrical conductivity of 0.055 µS∙cm^−1^ at 25 °C) was used throughout the experiments.

### 2.2. Reduction in Graphene Oxide (rGO)

For reducing graphene oxide (GO), a similar procedure was applied to the one reported by Baptista-Pires et al. [31]. First, commercial GO water dispersion (4 mg∙mL^−1^ concentration) was diluted in water to a final concentration of 1 mg∙mL^−1^. Then, an equal volume ratio of prepared GO solution was mixed with a 1 mg∙mL^−1^ ascorbic acid solution, followed by a thermal treatment in the microwave (MW) oven (Microwave Reaction System SOLV, Multiwave PRO, Anton-Paar GmbH, Graz, Austria) at 125 °C for 45 min. The material was collected in a 500 mL Erlenmeyer flask, filtered, and washed several times with deionized water. Finally, the material was dried overnight at 65 °C and labeled as reduced graphene oxide (rGO).

### 2.3. N/TiO_2_/rGO Microwave-Assisted Synthesis

For N/TiO_2_/rGO synthesis, the sol–gel method was combined with the microwave-assisted approach, varying the amount of rGO (0.25–10 wt.%). Firstly, a certain amount of rGO was dispersed in ethanol and sonicated for 45 min at 35 Hz. Meanwhile, TTIP was mixed with AcAc and stirred for several minutes, then ethanol/rGO solution was added while stirring at room temperature; these reagents were mixed at a molar ratio of TTIP:AcAc:EtOH = 0.014:0.039:1.37 and labeled as solution A. On the other hand, urea (N/Ti molar ratio equal to 12) was dissolved in 20 mL of deionized water and labeled as solution B. Then, solutions A and B were added dropwise to 80 mL of deionized water while stirring at room temperature. This final solution was kept under stirring conditions for 1 h at room temperature. Then, the solution was transferred to four Teflon vessels in the microwave (MW) oven (Microwave Reaction System SOLV, Multiwave PRO, Anton-Paar GmbH, Graz, Austria) for thermal treatment at 200 °C and 10 min. Inner pressure and temperature were monitored during the synthesis process using a *p*/*T* sensor accessory (Anton-Paar GmbH, Graz, Austria), as shown in the Supplementary Material (Figure S1). The synthesized material was washed several times with ethanol and water, centrifuged, and dried at 65 °C overnight. The obtained materials were labeled as N/TiO_2_/rGO x wt.%, where x represents the amount of rGO added. For comparison purposes, N/TiO_2_ material was synthesized using the same procedure and quantities described above but without dispersing rGO in ethanol.

### 2.4. Characterization of Photocatalysts

The BET surface area, pore volumes, and pore size distribution were calculated from nitrogen adsorption and desorption isotherm data using an ASAP 2000 apparatus (Micromeritics Corporation, Norcross, GA, USA). Prior to analysis, the sample was degassed under a dynamic vacuum of 6.6 mPa at 150 °C for 10 h.

Fourier transform infrared spectroscopy (FTIR) spectra were recorded on an IRSpirit (Shimadzu, Kyoto, Japan) in the range of 400–4000 cm^−1^, using an attenuated total reflection accessory.

Raman measurements were performed by confocal micro-Raman spectroscopy using a T64000 (Horiba Jobin Yvon, Kyoto, Japan), equipped with a solid-state laser with a wavelength of 532.5 nm and a 50× *g* magnification and large working distance objective in the range of 90–3000 cm^−1^. Possible TiO_2_ phase transition during measurement was avoided by laser power adjustment.

An XRD6000 (Shimadzu, Kyoto, Japan) X-ray diffractometer with CuKα radiation was used for XRD analysis. The fixed-step scans were collected in the 2θ range of 15–80° with steps of 0.02° 2θ and a counting time of 0.6 s under an accelerating voltage of 40 kV and a current of 30 mA.

Energy bandgap (*E_g_*) was calculated from diffuse reflectance spectroscopy (DRS) measurements, which were performed on a QE Pro High-Performance Spectrometer (Ocean Insight, Orlando, FL, USA) equipped with an integrating sphere and a DH 2000 deuterium–halogen source in the analysis range 200–1000 nm with a resolution of 1 nm and integration time of 10 s.

Elemental surface composition was determined by scanning electron microscopy (SEM) using a Vega Easyprobe 3 device (Tescan, Brno, Czech Republic). Energy-dispersive spectroscopy (EDS) spectra were recorded with an XFlash 6|30 detector (Bruker, Billerica, MA, USA) at a working distance of 10 mm under an accelerating voltage of 20 keV.

The chemical composition and energy binding were determined by an XPS spectrometer equipped with a Phoibos MCD 100 electron analyzer (SPECS, Berlin, Germany) and a monochromatic source of Al Kα X-rays of 1486.74 eV. During analysis, the typical pressure in the UHV chamber was in the 10^−7^ Pa range. For the electron pass energy of the hemispherical electron energy analyzer of 10 eV used in the present study, the overall energy resolution was around 0.8 eV. All spectra were calibrated by the position of C 1s peak, placed at the binding energy of 284.5 eV. The XPS spectra were deconvoluted into several sets of mixed Gaussian–Lorentzian functions with Shirley background subtraction.

### 2.5. Adsorption, Photolytic and Photocatalytic Experiments

Initially, the photocatalytic activity of N/TiO_2_ and N/TiO_2_/rGO nanocomposites with 0.25, 1, 3, 5, and 10 wt.% of rGO was evaluated through the degradation of ciprofloxacin (CIP) using different radiation sources: a UVA lamp, model UVAHAND LED (Dr. Hönle AG, UV-Technologie, Gilching, Germany) (peak on 365 nm, 70 W), solar light simulator (SLS) model SOL500 (Dr. Hönle AG, UV-Technologie, Gilching, Germany) (430 W), cold visible light (CVL), and a model OSRAM Endura Flood 100 W 840 GD (Ledvance GmbH, Osram, Munich, Germany) (450 nm and 600 nm, 100 W). At the same time, the commercial photocatalyst TiO_2_ P25 (Degussa AG, Germany) was used as reference material for photocatalytic activity. Prior to the photocatalytic test, the adsorption–desorption equilibrium was determined. For the adsorption process, 25 mg of the photocatalyst was dispersed in 100 mL of pollutant solution (10 mg∙L^−1^) and left in the dark for 2 h. Samples were taken at different time intervals, filtered using a 0.45 µm mixed cellulose ester membrane filter, and directly analyzed with a UV-Vis spectrophotometer (HEWLETT PACKARD, Model HP 8430, Palo Alto, CA, USA) at 273 nm (maximum absorption peak of CIP).

For a photocatalytic test, in each experiment, 25 mg of the photocatalyst was dispersed in 100 mL of pollutant solution (10 mg∙L^−1^) and irradiated from above with lamps 20 cm away from the reactor. Before irradiation, the suspension was stirred for 30 min in the dark to ensure adsorption–desorption equilibrium, which had been determined previously by the adsorption test. After that, the lamp was turned on, and the suspension was irradiated for 2 h. Samples were taken from the reactor at intervals (0, 5, 10, 20, 30, 45, 60, 90, and 120 min), filtered using a 0.45 µm mixed cellulose ester membrane filter, and directly analyzed by high liquid chromatography (HPLC, SCL-10A, Shimadzu) equipped with UV-Vis detector (SPD-10 AV, Shimadzu) at 273 nm. The separation was carried out on a column Shim-pack VP-ODS (4.6 × 150.0 mm; 5 μm, Shimadzu). The mobile phase was a mixture of 2% acetic acid/acetonitrile (*v*/*v*, 84:16) with a 1 mL∙min^–1^ flow rate in the isocratic mode. During the photocatalytic experiment, the temperature was kept at 25 °C by a thermostatic bath.

For the N/TiO_2_/rGO material that showed higher photocatalytic activity in the degradation of CIP under different radiation sources, additional adsorption and photocatalytic activity tests were carried out by the degradation of diclofenac (DCF, 10 mg∙L^−1^) and salicylic acid (SA, 10 mg∙L^−1^), using the same radiation sources mentioned above, and using an additional source of light: blue visible light (BVL) lamp, model UVAHAND LED (Dr. Hönle AG, UV-Technologie, Gilching, Germany) (peak on 405 nm, 70 W). The same experimental methodology mentioned above was applied. However, for the adsorption test, the samples were analyzed in the UV-Vis spectrophotometer at 276 nm (maximum absorption peak of DCF) and 297 nm (maximum absorption peak of SA). Meanwhile, for the photocatalytic test, the separation of DCF and SA was achieved using a mobile phase mixture of 2% phosphoric acid/acetonitrile (*v*/*v*, 40:60) with a flow rate of 1 mL∙min^−1^ in the isocratic model. For the photolysis process, 100 mL of pollutant solution (CIP, DCF, and SA, 10 mg∙L^−1^) without a catalyst was irradiated for 2 h, applying the same sampling and analysis methods described above for the photocatalytic test. For every adsorption and photocatalytic test, the pH value was measured at the beginning and the end of each experiment using a pH meter-type Sevencompact pH/Ion S220 (Mettler-Toledo Co., Columbus, OH, United States). For photocatalytic mechanism determination, a similar procedure as the photocatalytic test was performed, but which differed by the addition of the scavenger agents such as methanol (°OH ), p-Benzoquinone (O_2_°¯), and formic acid (*h*+) prior to the photocatalytic test.

In addition to the photocatalytic mechanism determination, the irradiation intensities effect of each lamp in the degradation of CIP was evaluated, placing each lamp at two different heights from the photocatalytic reactor. Prior to this analysis, global and UV-A irradiation intensities of each lamp were measured at different heights. Global irradiation was measured using a pyranometer in the range 285–2800 nm (Kipp & Zonen Co., model CMP11, Delft, The Netherlands), while the UV-A irradiation was measured by a radiometer equipped with a UV-A sensor in the range 315–400 nm (Opsytec Dr. Gröbel Co., model RM 21, Ettlingen, Germany). Global and UV-A irradiation charts of each lamp (Figure S2) and radiation spectra of the different lamps used (Figure S3) are presented in the Supplementary Material.

## 3. Results & Discussion

### 3.1. Reduction in Graphene Oxide

**Figure 1:**
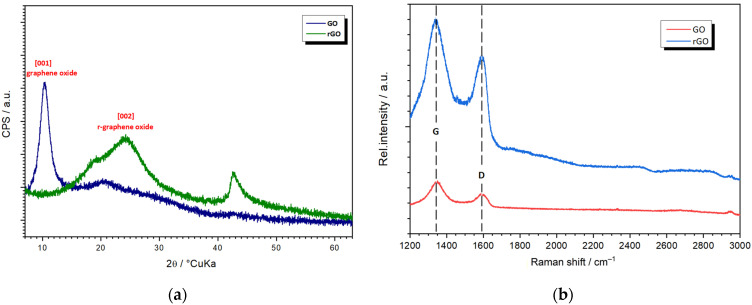
(**a**) X-ray diffraction patterns, and (**b**) Raman spectra of GO and rGO.

XRD and Raman analyses were performed to confirm the reduction in graphene oxide. The XRD results for GO and rGO are presented in Figure 1a. The GO sample has a diffraction peak at 2θ = 10.4°, characteristic of the (001) plane, which arises in GO due to oxygenated functional groups on the carbon. In the rGO sample, the absence of the diffraction peak at the maximum related to the (001) plane, and the appearance of a diffraction peak at 2θ = 24.3°, characteristic of the (002) plane, suggests the formation of reduced graphene oxide (rGO) [32]. Another diffraction peak for rGO appears at 2θ = 42.7°, which could be attributed to stack disorder layers of rGO [33].

The Raman analysis shows that both samples (GO and rGO) have the characteristic G and D bands, around 1350 and 1590 cm^−1^, respectively (Figure 1b). The G and D bands are typical for all sp^2^ carbon structures. Usually, the D band is associated with the breathing mode of sp^3^ defects in the carbon structure. In contrast, the G band refers to stretching vibrations of ordered sp^2^-bonded carbon atoms in a two-dimensional hexagonal lattice [34]. In the GO sample, the D band appears at 1350 cm^−1^, while for the rGO, this band was shifted to lower wavenumber (1342 cm^−1^). This shift of the D band on the rGO is probably due to the reduction in the size of in-plane sp^2^ domains by creating defects, vacancies, and distortions of the sp^2^ domains after complete reduction [35]. On the other hand, the G bands for the GO and rGO are found at 1590 cm^−1^ and 1591 cm^−1^, respectively. The slight shifting to a higher wavenumber in the rGO could be due to the removal of oxygen moieties [33]. Based on the XRD and Raman analysis, it has been determined that the reduction in graphene oxide was achieved under mild conditions using non-hazardous chemicals.

### 3.2. Characterization of Microwave-Assisted Synthesized N/TiO_2_/rGO Nanocomposites

Nitrogen adsorption/desorption isotherms were used to investigate the Brunauer, Emmett, and Teller (BET) specific surface area. The pore size distribution was determined by the Barrett–Joyner–Halenda (BJH) method (Supplementary Material, Figure S4). The specific surface area, pore volume, and average pore diameter of N/TiO_2_, N/TiO_2_/rGO, P25 photocatalysts, and rGO material are presented in Table 1. All analyzed materials exhibit a large specific surface area compared to the commercial photocatalysts P25 (48 m^2^∙g^−1^).

All materials display type IV nitrogen adsorption–desorption isotherms, typical for mesoporous materials, with an H2 hysteresis type, indicating that the porosity is composed mainly of neck-like and wide-body pores (Supplementary Material, Figure S5) [36,37]. On the other hand, the introduction of rGO changed porosity, as the specific surface area of N/TiO_2_/rGO materials is increased, while pore diameter is slightly decreased compared to N/TiO_2_ material. However, changes in porosity can be observed regardless of the amount of rGO, indicating that the amount of rGO that could be incorporated into the photocatalyst is limited.

**Figure 2:**
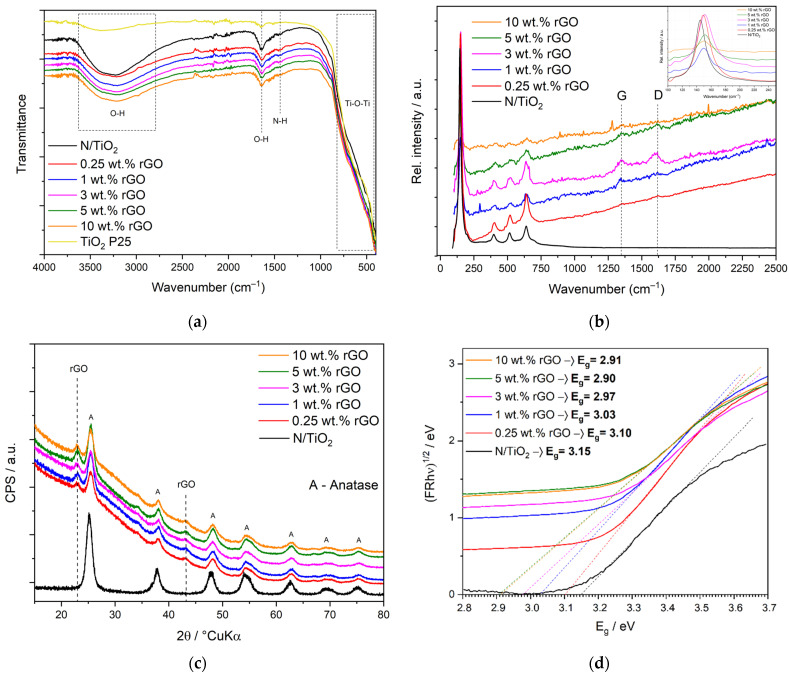
(**a**) FTIR spectra, (**b**) Raman spectra, (**c**) X-ray diffraction patterns, and (**d**) Tauc plot for energy bandgap determination of N/TiO_2_ and N/TiO_2_/rGO photocatalysts with different amounts of rGO (0.25, 1, 3, 5 and 10 wt.%).

Figure 2a presents the FTIR spectra of N/TiO_2_ and N/TiO_2_/rGO materials. All photocatalysts (N/TiO_2_ and N/TiO_2_/rGO) show a strong and wide band between 400 and 800 cm^–1^, which is usually associated with the stretching of vibrations of Ti–O–Ti bonds [38]. Furthermore, the region between 3670 and 2800 cm^−1^ is correlated to the stretching of vibrations of the O–H group, while the peak at 1638 cm^−1^ corresponds to the stretching of vibrations of the O–H group related to adsorbed water [39]. On the other hand, N/TiO_2_ and N/TiO_2_/rGO photocatalysts contain up to 3 wt.% rGO. A small peak around 1455 cm^−1^ is observed, which could be attributed to the stretching of vibrations of the N–H group [40]. Although no band associated with rGO was found, all N/TiO_2_/rGO materials present a reduction in the intensity of the bands related to the O–H group (3670–2800 cm^−1^ and 1638 cm^−1^), which could be associated with the hydrophobic effect of rGO.

The Raman spectra of N/TiO_2_ and N/TiO_2_/rGO materials are displayed in Figure 2b. For all materials, four peaks are observed at 144 cm^−1^(E_g_), 398 cm^−1^(B_1g_), 515 cm^−1^(A_1g_), and 637 cm^−1^(E_g_) which were associated with Raman modes of the anatase phase of TiO_2_. Peaks related to the rutile phase of TiO_2_ were not detected in the synthesized materials. Additionally, two peaks at 1350 cm^−1^ and 1618 cm^−1^ associated with the disorder (G band) and graphitic carbon (D band) of rGO are observed in materials containing different amounts of rGO. In the N/TiO_2_ material, the characteristic G and D bands of rGO are not detected. The E_g_ Raman mode at 144 cm^−1^ is shifted to a higher wavenumber (149 cm^−1^) when rGO is incorporated, probably due to the surface defects created by the rGO, which could indicate a good interaction between the rGO and TiO_2_ that could promote the charge separation during the photocatalytic process [41,42].

Figure 2c shows the X-ray diffraction (XRD) patterns of N/TiO_2_ and N/TiO_2_/rGO materials. The XRD analysis reveals that all synthesized materials present diffraction peaks at 25.40° (101); 38.08° (112); 48.15° (200); 55.07° (211); 62.88° (204); 70.31° (220); 75.03° (215) related to the anatase phase (ICDD PDF#21-1272) [19]. In addition, two diffraction peaks around 23° and 43° 2θ could be related to rGO and the stack disorder layers of rGO, respectively, which are similar to the peaks previously identified in Figure 1a.

Figure 2d shows the Tauc plots of N/TiO_2_ and N/TiO_2_/rGO materials, where the energy bandgap is determined from the diffuse reflectance spectroscopy (DRS). It is observed that all N/TiO_2_/rGO materials present lower energy bandgap in comparison to N/TiO_2_ and commercial TiO_2_ P25 (3.20 eV) [19]. Additionally, it is noticed that an increase in the amount of rGO decreases the energy bandgap. However, for loading values beyond 5 wt.% rGO, no additional decrease in the energy bandgap was observed. The Tauc plot results could indicate that apart from the electron trap effect known in the rGO, it can also act as an energy bandgap narrower.

The elemental composition of N/TiO_2_ and N/TiO_2_/rGO materials determined by SEM-EDS analysis are presented in Table 2. The compositional analysis shows that carbon composition increases, and oxygen composition decreases, when the amount of rGO is incremented, which could be attributed to the lower number of oxygen functional groups in the rGO structure achieved during the reduction process of GO. Additionally, it is observed that the highest carbon moieties see around 3–5 wt.% of rGO be achieved. The nitrogen element is not detected in any N/TiO_2_/rGO materials. This may easily be the consequence of large probing depth and volume for the EDS method and general coverage of nitrogen moieties by carbon moieties. EDS analysis is not a precise quantitative method, especially for light elements like nitrogen and carbon; therefore, additional techniques such as XPS analysis are helpful to complement surface characterization.

Table 3 shows the bulk composition of N/TiO_2_ and N/TiO_2_/rGO with 0.25 wt.% photocatalysts, determined by XPS analysis. The bulk analysis shows a drop in the oxygen fraction in the N/TiO_2_/rGO photocatalyst compared to N/TiO_2_, which could be attributed to the reduction in oxygen functional groups of rGO. The carbon fraction is also higher in the N/TiO_2_/rGO due to rGO content, while in the N/TiO_2_ material is attributed to impurities from the synthesis (urea o acetylacetone). The elemental analysis by XPS is more accurate than SEM-EDS, the latter one being used as an estimation.

**Figure 3:**
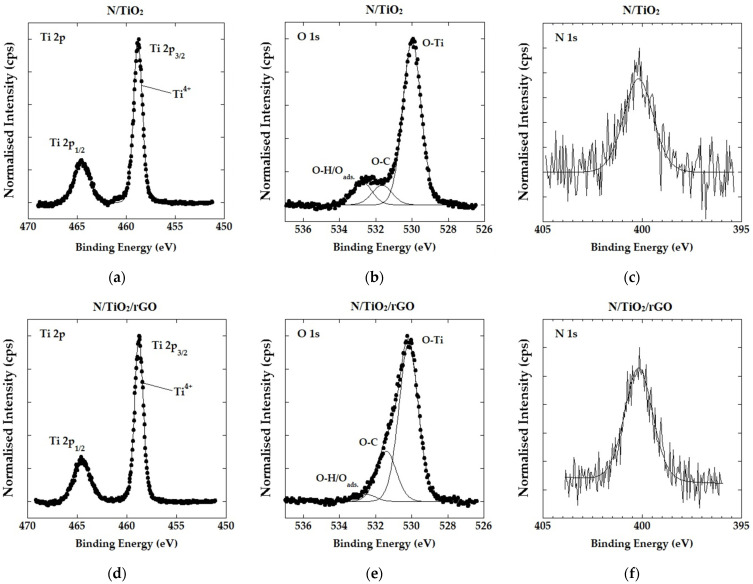
High-resolution XPS spectra of N/TiO_2_ and N/TiO_2_/rGO with 0.25 wt. % of rGO. (**a**–**c**) are the Ti 2p spectrum, O 1s spectrum, and N 1s spectrum, respectively, for N/TiO_2_. (**d**–**f**) are the Ti 2p spectrum, O 1s spectrum, and N 1s spectrum, respectively, for N/TiO_2_/rGO.

Figure 3 shows the high-resolution Ti, O, and N spectra obtained by XPS analysis for N/TiO_2_ and N/TiO_2_/rGO with 0.25 wt.% of rGO are shown, in which the chemical composition and oxidation states were determined. In Figure 3a,d, the Ti 2p spectra for N/TiO_2_ and N/TiO_2_/rGO with 0.25 wt.% of rGO are shown, respectively. In both samples, the formation of Ti^4+^ related to the anatase phase of TiO_2_ is confirmed by the detection of two signals, Ti 2p_1/2_ and Ti 2p_3/2_, at 458.7 eV and 464.5 eV, respectively [43]. The O 1s spectra for N/TiO_2_ and N/TiO_2_/rGO with 0.25 wt.% of rGO are shown in Figure 3b,e. In both samples, three binding energies are detected, where the main peak associated with oxygen bonded to titanium (O-Ti) is located at 530.2 eV. A second peak, located at 532.8 eV, corresponds to O-H bonds that could be attributed to the surface hydroxylation by chemisorbed water. It can be noticed that this peak has a lower intensity in N/TiO_2_/rGO, with 0.25 wt.% of rGO, than N/TiO_2_ material due to the hydrophobic nature of rGO [44]. On the other hand, the third peak is detected at 531.6 eV in the N/TiO_2_/rGO with 0.25 wt.% of rGO being associated with oxygen bonded to carbon (O-C). This could be attributed to the binding between the carbon of rGO and the oxygen atom of the TiO_2_ structure or due to oxygenated groups that were not completely removed during the reduction process [45]. Meanwhile, the oxygen bonded to carbon (O-C) with less intensity detected in the N/TiO_2_ material at 531.6 eV could be related to impurities from the synthesis (urea o acetylacetone). The changes in peak intensity at 532.8 eV and 531.6 eV on N/TiO_2_/rGO with 0.25 wt.% of rGO, compared to the N/TiO_2_ material, could indicate that rGO was successfully incorporated into the TiO_2_ structure. Figure 3c,f display the N 1s spectra for N/TiO_2_ and N/TiO_2_/rGO with 0.25 wt.% of rGO, respectively. In both samples, the binding energy is found to be above 400 eV, an observation which indicates that nitrogen is located in the interstitial voids rather than substituting the oxygen atoms [46]. Based on that, the energy binding at 400.15 eV could be assigned to nitrogen bonded to oxygen (N-O) [47]. Some studies have shown that N-O bonding in the interstitial sites creates new energetic levels that contribute to the absorption in the visible light range without modifying the energy bandgap [48,49], as observed in Figure 2d (N/TiO_2_). Additionally, it is observed that the electronic densities of Ti and N remain unaltered after the incorporation of rGO. On the other hand, the electronic density of O is slightly shifted to higher binding energy from 529.95 eV to 530.25 eV, probably due to the bonding of carbon (rGO) and oxygen from TiO_2_.

### 3.3. Photocatalytic Performance of Microwave-Assisted Synthesized N/TiO_2_/rGO Nanocomposites

**Figure 4:**
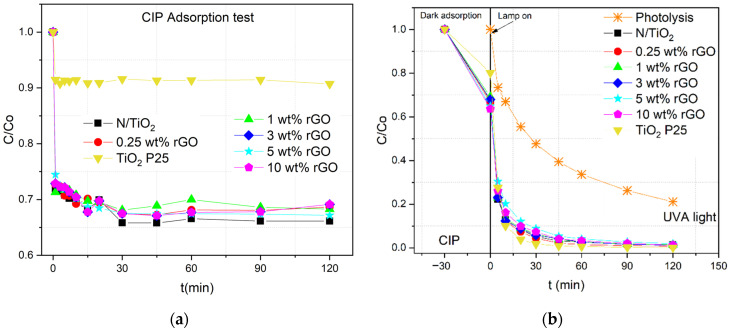

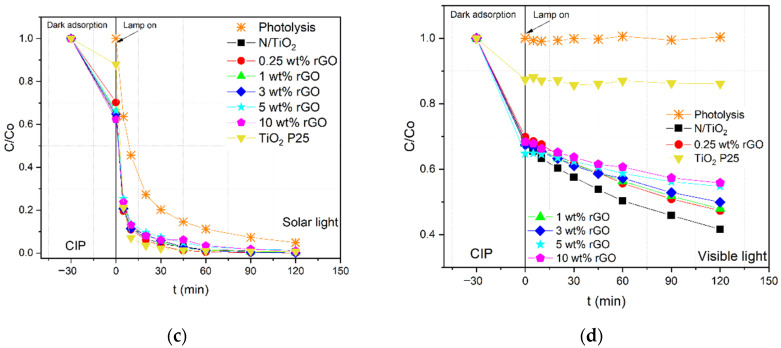
(**a**) Adsorption–desorption equilibrium and photocatalytic degradation of ciprofloxacin CIP by N/TiO_2_ and N/TiO_2_/rGO with different amounts of rGO under (**b**) UVA light, (**c**) solar light simulator (SLS), and (**d**) cold visible light (CVL).

The photocatalytic activity of N/TiO_2_, N/TiO_2_/rGO materials, and commercial TiO_2_ P25 was evaluated through the degradation of CIP aqueous solution (10 mg∙L^−1^) under ultraviolet A light (UVA), solar light simulator (SLS) and cold visible light (CVL), as shown in Figure 4. UV/Vis absorbance spectra of the initial concentration of CIP are shown in the Supplementary Material, Figure S6a. Before the photocatalytic test, the adsorption–desorption equilibrium was determined, as shown in Figure 4a. The initial pH of the CIP solution was in the range of 6.2–6.5; after the adsorption test, the pH values were kept the same. It is observed that N/TiO_2_ and N/TiO_2_/rGO materials remove around 30% of CIP just by adsorption process (Table 4), while commercial TiO_2_ P25 removes only 10% of CIP by adsorption, probably because of the differences in the specific surface area. Although N/TiO_2_/rGO materials have a 20% higher specific surface area than N/TiO_2_ material (Table 1), there is no significant difference in the adsorption capacities of the synthesized materials. Additionally, fast pollutant adsorption onto the surface of photocatalysts is noticed, achieving the adsorption–desorption equilibrium in less than 30 min. The fast adsorption could be due to the high affinity of the amine and carboxylic groups of CIP with the oxygen functional groups of materials that favor the adsorption by hydrogen bonds.

From the photocatalytic experiments, it is observed that under UVA and SLS conditions (Figure 4b,c), all the materials show similar behavior, whereby after 20 min of irradiation, more than 90% of CIP degradation is achieved. After 60 min of irradiation, no significant degradation is observed. On the contrary, the photocatalytic experiments under CVL (Figure 4d) show that none of the synthesized materials achieve a complete CIP removal; even after 120 min of irradiation, only 50% of CIP removal is obtained; commercial TiO_2_ P25 does not exhibit any photocatalytic activity, as expected. Under the three different light sources, it is observed that the CIP removal is accomplished by a synergistic effect of adsorption and photodegradation processes. Additionally, regardless of the irradiation source, photocatalytic activity decreases when the rGO load increases, indicating the rGO:TiO_2_ ratio of 0.25 wt.% of rGO is the most suitable dosage load. In general, an excess of the dosing element has a negative effect on photocatalytic properties [50], and considering that the material has already been doped with nitrogen at this point in the process, the rGO amount required for dosing is probably quite low. An excess of rGO load could diminish the photocatalytic activity, mainly for two reasons. The first is the generation of a shielding effect, where the rGO excess could block the light, reducing the probability that TiO_2_ could absorb photons. The other reason may be that the excess rGO could act as a recombination center instead of preventing the recombination, depleting the photoactivity [51,52,53]. Besides photocatalytic degradation, CIP is susceptible to photolytic decomposition by UVA and SLS irradiations. However, the CIP degradation rates by photolysis are much lower than the photocatalytic degradations under UVA and SLS, as observed in Table 4. In the case of CVL, no photolytic degradation for CIP is detected. After photocatalytic tests, the pH values were around 6.4–6.8, a result probably caused by the formation of by-products with less-acidic functional groups.

In the case of N/TiO_2_/rGO nanocomposite with 0.25 wt.% of rGO, the photocatalytic experiments under UVA and SLS show that the introduction of rGO improves the degradation rate compared to N/TiO_2_, and under SLS, its photoactivity is comparable to the commercial TiO_2_ P25. Under CVL radiation, the degradation rate is generally reduced, probably due to part of the nitrogen being covered by rGO, as was noticed in the SEM-EDS compositional analysis. These results indicate that the rGO effectively acts as a sink for electrons in the composite, reducing the recombination of electron/hole charge pairs [24]. On the other hand, comparing the degradation rate between UVA and SLS lamps, it is noticed that the photocatalytic activity is improved under solar irradiation conditions, despite the fact that the SLS lamp has a lower UV-A radiation intensity (59.2 W∙m^−2^) than the UVA lamp (98.5 W∙m^−2^), something which will be discussed in more detail at the end of this subchapter. This photocatalytic activity improvement under solar radiation could be attributed to the light absorption shifting to the visible light range. As confirmed by XPS analysis, the introduction of nitrogen in the interstitial voids of the composite creates new energetic levels that allow photons to be absorbed from wavelengths higher than 365 nm [17,54]. Therefore, the co-doping of TiO_2_ with nitrogen and rGO seems to be a promising approach for improving the overall photocatalytic activity because it shifts the photoresponse to the visible light range and reduces charge recombination at the same time.

For the photocatalytic tests, the degradation rate is analyzed using the pseudo-first-order and pseudo-second-order models to identify which model better describes the degradation process. The pseudo-first-order models represents a degradation mechanism affected mainly by the changes in the pollutant concentration, while in the pseudo-second-order model, several factors, such as light intensity, pollutant concentration, by-product formations, etc., define the degradation rate. The kinetic constant for the pseudo-first-order model is obtained by the slope of the plot—Ln(*C/Co*) versus the irradiation time. For the pseudo-second-order, the kinetic constant is obtained by the slope of the plot (1/*C*–1/*Co*) versus the irradiation time. Based on the parameters fitting, it could be determined which model explains better the degradation mechanism. Table 4 shows the pseudo-first-order (*k*_1_, min^−1^) and pseudo-second-order (*k_2_*, L∙mg∙min^−1^) kinetic constants, their correlation coefficients (*R*^2^), and efficiencies for the CIP removal by N/TiO_2_, N/TiO_2_/rGO materials, and commercial TiO_2_ P25. Under UVA and SLS, it is noticed that the pseudo-second-order model has an *R*^2^ > 0.90, while the pseudo-first-order model has a lower correlation coefficient (*R*^2^ < 0.90). In the case of CIP removal under CVL, *R*^2^ is higher than 0.90 in both models; however, the pseudo-second-order model better fits the data. Thus, the CIP removal follows a pseudo-second-order model, where the CIP removal is defined by other factors and not just by the changes in its concentration.

**Table 4:** Pseudo-first-order and pseudo-second-order kinetic parameters and efficiencies of CIP removal by N/TiO_2_, TiO_2_ P25, and N/TiO_2_/rGO materials under UVA, solar light simulator (SLS), and cold visible light (CVL).

Lamp	Material	Removal by Adsorption	Removal Efficiency (%)	Model			
				Pseudo-First Order		Pseudo-Second Order	
		(%)	*ɳ*	*k*_1_	*R* ^2^	*k*_2_	*R* ^2^
UVA	N/TiO_2_	32.44	98.02	0.0432	0.8635	0.5881	0.9975
	TiO_2_ P25	19.87	99.58	0.0589	0.8508	1.9217	0.9937
	N/TiO_2_/rGO 0.25 wt.%	33.47	98.81	0.0489	0.8801	0.9388	0.9910
	N/TiO_2_/rGO 1 wt.%	30.87	97.95	0.0425	0.8710	0.5652	0.9976
	N/TiO_2_/rGO 3 wt.%	32.15	98.21	0.0433	0.8782	0.6245	0.9928
	N/TiO_2_/rGO 5 wt.%	35.15	97.04	0.0379	0.9114	0.3992	0.9763
	N/TiO_2_/rGO 10 wt.%	36.40	98.01	0.0407	0.8985	0.5093	0.9844
	Photolysis	*	78.92	0.0154	0.9404	0.0028	0.9953
SLS	N/TiO_2_	36.32	98.77	0.0477	0.8843	0.9768	0.9869
	TiO_2_ P25	12.04	99.26	0.0558	0.8122	1.2612	0.9839
	N/TiO_2_/rGO 0.25 wt.%	29.75	99.90	0.0531	0.8645	1.2462	0.9866
	N/TiO_2_/rGO 1 wt.%	33.96	99.42	0.0468	0.8773	0.8664	0.9873
	N/TiO_2_/rGO 3 wt.%	35.12	99.82	0.0446	0.8869	0.7629	0.9861
	N/TiO_2_/rGO 5 wt.%	33.59	97.88	0.0410	0.8879	0.4908	0.9921
	N/TiO_2_/rGO 10 wt.%	37.70	98.34	0.0404	0.8870	0.5094	0.9771
	Photolysis	*	95.10	0.0304	0.9279	0.0140	0.9922
CVL	N/TiO_2_	31.20	58.36	0.0046	0.9832	0.0079	0.9949
	TiO_2_ P25	*	*	*	*	*	*
	N/TiO_2_/rGO 0.25 wt.%	30.21	52.69	0.0035	0.995	0.0052	0.9991
	N/TiO_2_/rGO 1 wt.%	32.15	52.10	0.003	0.9983	0.0050	0.9995
	N/TiO_2_/rGO 3 wt%	32.54	50.07	0.0027	0.9937	0.0044	0.9977
	N/TiO_2_/rGO 5 wt.%	35.27	45.20	0.0015	0.9897	0.0021	0.9898
	N/TiO_2_/rGO 10 wt.%	31.70	44.17	0.0019	0.9860	0.0026	0.9904
	Photolysis	*	*	*	*	*	*

* No photolytic or photocatalytic degradation was observed.

**Figure 5:**
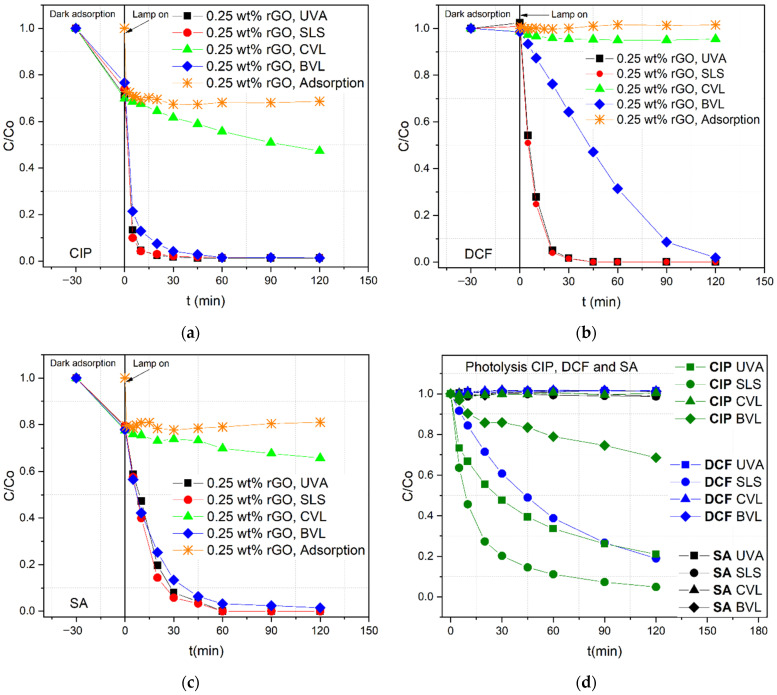
Adsorption and photocatalytic degradation of (**a**) ciprofloxacin (CIP), (**b**) diclofenac (DCF), and (**c**) salicylic acid (SA) by N/TiO_2_/rGO with 0.25 wt.% of rGO material under UVA light, solar light simulator (SLS), cold visible light (CVL), and blue visible light (BVL). (**d**) Photolysis of CIP, DCF, and SA under different irradiation sources.

After determining that the lowest load amount of rGO (N/TiO_2_/rGO with 0.25 wt.% of rGO) is the most photoactive nanocomposite, its photocatalytic activity was further evaluated in the degradation of diclofenac (DCF, 10 mg∙L^−1^) and salicylic acid (SA, 10 mg∙L^−1^) under different radiation sources, including an additional light source (Blue visible light (BVL)), the results of which are presented in Figure 5. UV/Vis absorbance spectra of the initial concentration of DCF and SA are shown in the Supplementary Material, Figure S6b,c, respectively. The initial pH of the DCF solution was around 5.3–5.5, while the initial pH of SA was in the range of 4.1–4.2. For the photocatalytic degradation of CIP and DCF under UVA and SLS, it is noticed that just 20 min of irradiation is needed to achieve more than 90% of pollutant removal. For SA, at least 30 min is required to complete more than 90% removal under the same irradiation sources. However, after 60 min of irradiation under UVA and SLS conditions, all three OMPs have been almost completely degraded. In the case of photocatalytic degradation under BVL, CIP and SA conditions follow similar degradation behavior as under UVA and SLS exposure, where no noticeable change is detected after 60 min of irradiation. However, the DCF requires 120 min of irradiation to obtain similar removal efficiencies as under UVA and SLS. On the contrary, when the photocatalyst is irradiated with CVL, very low photocatalytic activity for removal of SA is detected, while for the DCF, the photodegradation can be neglected completely. After photocatalytic tests, the pH values in both pollutants remained similar to the initial pH values.

In the case of CIP and SA, a synergistic effect of adsorption and photodegradation occurs, while for the DCF, the photocatalytic process is the only mechanism for pollutant removal. These results show that adsorption plays an important role in pollutant removal under low-energy irradiation sources (BVL and CVL). Although DCF and SA are negatively charged, and although CIP is positively and negatively charged (zwitterion form), under the study conditions the differences in the adsorption could be more related to the polarity of each molecule. CIP and SA are polar molecules, while DCF is a non-polar molecule. On the other hand, CIP is the most susceptible molecule to photolytic degradation, being degraded under BVL but to a lesser extent. DCF only is decomposed under SLS, probably by the most energetic fraction of solar radiation. On the contrary, SA is not affected by photolysis under any of the irradiation sources applied.

Table 5 shows the pseudo-first-order (*k*_1_, min^−1^) and pseudo-second-order (*k*_2_, L∙mg^−1^∙min^−1^) kinetic constants, correlation coefficients (*R*^2^), and efficiencies for the CIP, DCF, and SA removal by N/TiO_2_/rGO nanocomposite with 0.25 wt.% of rGO under different irradiation sources; meanwhile, Table 6 contains the same parameters for the CIP and DCF removal by the photolysis process.

From the kinetic parameters, it is observed that CIP and SA follow the pseudo-second-order, while DCF follows a pseudo-first order model. These results indicate that the adsorption process influences the degradation kinetics. Again, it is noticed that when N/TiO_2_/rGO with 0.25 wt.% of rGO is irradiated with SLS, the kinetic constants increase compared to UVA in all evaluated OMPs, which could be attributed to the synergic effect the co-doping has in the composite, where nitrogen shifts the photoresponse to a broader absorption range, while rGO traps electrons. Additionally, it is observed that the kinetic constants, apart from the dependency on the OMP type, also depend on the type of light (Table 5). To understand the light dependency, UVA light is used as a reference for comparison purposes because it is the most common irradiation source for activating TiO_2_ photocatalytic processes. For the CIP and SA removal under SLS, the degradation rates increase 1.3 and 2.2 times, respectively, compared to the degradation rates under UVA. Conversely no significant improvement is observed for DCF.

On the contrary, the degradation rates decrease for all OMPs when the photocatalytic process is carried out under less energetic irradiation sources (BVL and CVL). Under BVL, the degradation rates for DCF and SA removal are reduced 1.5 times compared to UVA irradiation; meanwhile, for CIP the degradation rate is around 2.2 times lower than those under a UVA lamp’s ration. Interestingly, under CVL irradiation, CIP and SA have similar correlation coefficients for the pseudo-first and pseudo-second kinetic order. This indicates that the relevance of irradiation is lower for the degradation rates under less energetic wavelength sources, like this CVL lamp is.

**Table 5:** Pseudo-first-order, pseudo-second-order kinetic parameters, and CIP, DCF, and SA removal efficiencies by N/TiO_2_/rGO 0.25 wt.% photocatalyst under UVA, solar light simulator (SLS), cold visible light (CVL), and blue visible light (BVL).

Pollutant	Lamp	Removal Efficiency (%)	Model			
			Pseudo-First Order		Pseudo-Second Order	
		*ɳ*	*k*_1_	*R* ^2^	*k*_2_	*R* ^2^
CIP	UVA	98.81	0.0489	0.8801	0.9388	0.9910
	SLS	99.90	0.0531	0.8645	1.2462	0.9866
	CVL	52.69	0.0035	0.9950	0.0052	0.9991
	BVL	98.51	0.0425	0.8228	0.4585	0.9936
DCF	UVA	98.29	0.0403	0.9632	1.3493	0.9516
	SLS	98.68	0.0430	0.9669	1.7313	0.9349
	CVL	*	*	*	*	*
	BVL	91.08	0.0276	0.9490	0.7612	0.6573
SA	UVA	99.46	0.0344	0.9333	1.1296	0.9725
	SLS	99.28	0.0432	0.9404	2.4893	0.9589
	CVL	34.24	0.0016	0.9700	0.0084	0.9748
	BVL	96.15	0.0304	0.9313	0.7479	0.9839

* No photocatalytic activity.

It seems that the changes in the kinetic constants somehow could be determined by the energy emitted by the lamp and by the fact that the adsorption process takes place. Under the SLS radiation conditions, N/TiO_2_/rGO photocatalyst absorbs photons from the visible light range besides the UV-A region, which could produce a higher amount of ROS capable of degrading the pollutants. Moreover, the adsorbed contaminants can also be degraded by surface charges (*h*+/*e*−) apart from ROS. Besides, under less energetic radiation sources such as BVL and CVL, the adsorption process plays an important role in pollutant removal. The degradation is probably a result of the redox process on the surface rather than ROS. In the case of CIP and SA, where the adsorption process occurs, pollutant removal is easily achieved under BVL, and some degradation is observed under CVL. Meanwhile, for DCF, slower degradation is observed under BVL, and no degradation is detected under CVL, probably due to a lack of adsorption over the photocatalyst.

**Table 6:** Pseudo-first-order, pseudo-second-order kinetic parameters, and efficiencies CIP and DCF removal by photolysis under UVA, solar light simulator (SLS), and blue visible light (BVL).

Pollutant	Lamp	Removal Efficiency (%)	Model			
			Pseudo-First Order		Pseudo-Second Order	
		*ɳ*	*k*_1_	*R^2^*	*k*_2_	*R^2^*
CIP	UVA	78.92	0.0154	0.9402	0.0028	0.9953
	SLS	95.10	0.0304	0.9279	0.0140	0.9922
	BVL	31.41	0.0035	0.9167	0.0004	0.9367
DCF	SLS	80.99	0.0146	0.9964	0.0032	0.9784

### 3.4. Photocatalytic Mechanisms

In order to determine the effect of the irradiation source and understand the role of the different ROS in the photocatalytic removal of CIP, DCF, and SA, photocatalytic experiments adding scavenger species were carried out. Methanol, p-Benzoquinone, and formic acid were used as hydroxyl radical (°OH), superoxide radical (O_2_°¯), and hole (*h*+) scavenger/interfering agents, respectively. The molar ratio pollutant/scavenger was 1/100, except for *p*-Benzoquinone, which was 1/10 due to its limitations on the analytical determination.

**Figure 6:**
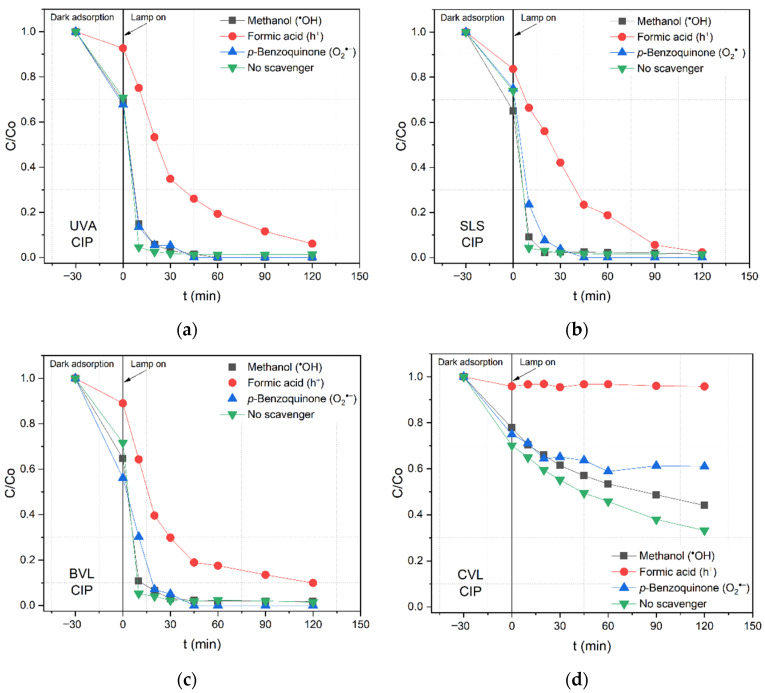
Photocatalytic degradation of ciprofloxacin (CIP) by N/TiO_2_/rGO with 0.25 wt.% of rGO material in the presence of different scavenger agents under (**a**) UVA light, (**b**) solar light simulator (SLS), (**c**) blue visible light (BVL), and (**d**) cold visible light (CVL).

Figure 6 shows the photocatalytic degradation of CIP by N/TiO_2_/rGO with 0.25 wt.% of rGO material in the presence of several scavenger agents under different irradiation sources. Overall, it is observed that under all irradiation sources, the addition of formic acid significantly reduces the degradation rate of CIP, indicating that the holes (*h*+) are the main oxidizing species involved in CIP removal. Additionally, it is noticed that, to a lesser extent, the addition of *p*-Benzoquinone and methanol has a slight reduction in the degradation rate of the CIP removal; however, under CVL, the contribution of these two scavenger agents becomes more significant. Moreover, it is observed that formic acid acts as an inhibitor in the CIP adsorption over the photocatalyst; this reduction in the CIP adsorption capacity is probably related to a higher affinity between the formic acid and the catalyst surface. Based on the results of CIP removal by N/TiO_2_/rGO with 0.25 wt.% of rGO material in the presence of several scavenger agents under different irradiation sources, it could be concluded that the photocatalytic mechanism is mostly independent of the irradiation wavelengths.

**Figure 7:**
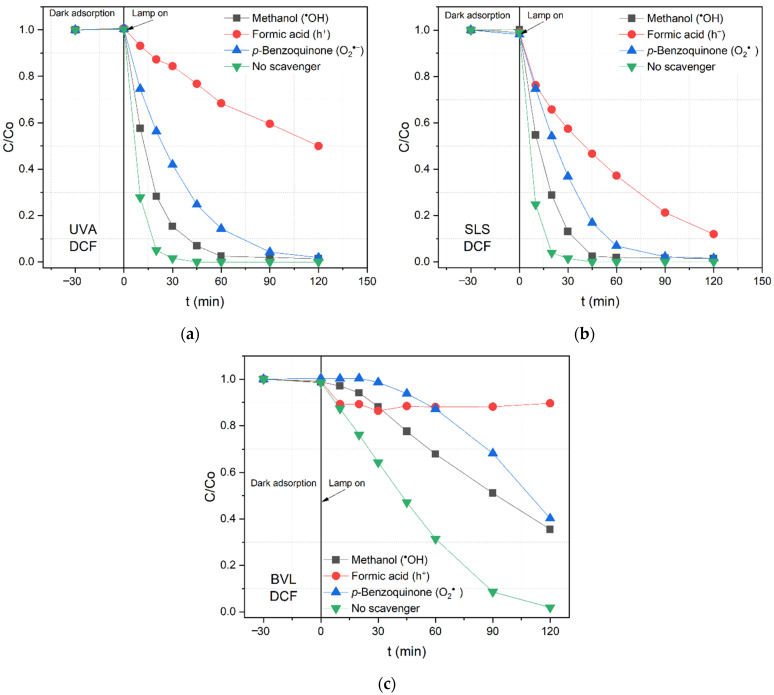
Photocatalytic degradation of diclofenac (DCF) by N/TiO_2_/rGO with 0.25 wt.% of rGO material in the presence of different scavenger agents under (**a**) UVA light, (**b**) solar light simulator (SLS), and (**c**) blue visible light (BVL).

The photocatalytic degradation of diclofenac (DCF) by N/TiO_2_/rGO with 0.25 wt.% of rGO material in the presence of scavenger agents under different irradiation sources is displayed in Figure 7. It is observed that formic acid diminishes the photocatalytic activity, indicating that the holes (*h*+) play a significant role in the DCF removal. However, in the removal of DCF, the role of superoxide (O_2_°¯) and hydroxyl radical (°OH) are also significant. Under BVL irradiation, it can be seen that DCF removal is not entirely achieved in any scavenging agent presence, indicating that all ROS are important for pollutant removal under less energetic wavelengths. Again, it is noticed that the photocatalytic mechanism remains independent of the irradiation wavelengths.

**Figure 8:**
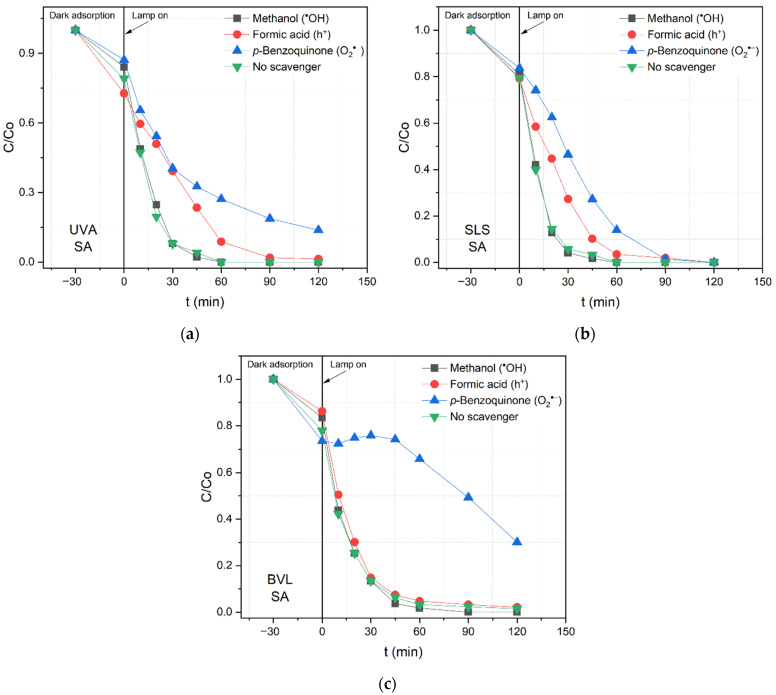
Photocatalytic salicylic acid (SA) degradation by N/TiO_2_/rGO with 0.25 wt.% of rGO material in the presence of different scavenger agents under (**a**) UVA light, (**b**) solar light simulator (SLS), and (**c**) blue visible light (BVL).

In the case of SA removal by N/TiO_2_/rGO with 0.25 wt.% of rGO material in the presence of different scavenger agents under different irradiation sources, as shown in Figure 8, the formic acid addition is a crucial oxidizing species. However, in the SA removal, the superoxide radical is the main oxidizing species, followed by the hole’s contributions. It is important to remark that, under high energetic wavelengths such as UVA and solar conditions, the adsorption of SA onto the photocatalyst surface favors the pollutant oxidation by the holes. Nevertheless, under the BVL source, the superoxide radical is only responsible for the SA removal, confirming that the photocatalytic mechanism does not rely on the irradiation wavelengths. Additionally, it is observed that the type of pollutant defines the photocatalytic degradation mechanism.

### 3.5. Irradiation Intensity Effect

Although the photocatalytic mechanism is not affected by the type of light (i.e., radiation) source, the degradation rate is light-source dependent, as was previously observed in the CIP, DCF, and SA removal under different irradiation wavelengths. Therefore, to determine the feasibility of using natural solar radiation to remove pharmaceuticals, the UVA and SLS lamps were adjusted to irradiation values of 16.5 W∙m^−2^ and 290 W∙m^−2^ of UV-A and global irradiation, respectively. Those irradiation values are similar to the average irradiation in the city of Zagreb, Croatia, during the spring–summer–autumn season, which is calculated based on the direct normal irradiation (global irradiation), taking the average hourly profiles between February and October [55]. The UVA and the SLS lamps were relocated at 50 and 60 cm away from the reactor, respectively, based on the irradiation chart measured for each lamp (Supplementary Material, Figure S1) to achieve the natural irradiation values. Based on a value of 290 W∙m^−2^ of global irradiation, BVL and CVL were adjusted to be 15 cm away from the reactor. Table 7 displays the global and UV-A irradiation values of each lamp at two different distances from the reactor.

**Figure 9:**
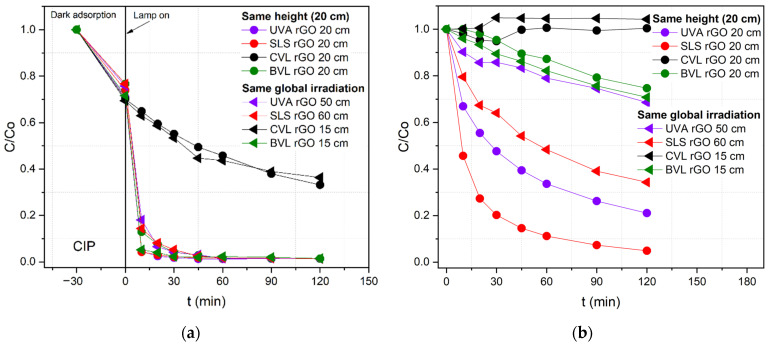
CIP removal by (**a**) photocatalyst N/TiO_2_/rGO with 0.25 wt.% of rGO and by (**b**) photolysis with lamps located at two different distances from the reactor.

CIP removal by photocatalysis and photolysis at different distances of the lamps from the reactor is displayed in Figure 9. From the photocatalytic process (Figure 9a), in which N/TiO_2_/rGO with 0.25 wt.% of rGO material is used, there is no evidence of significant changes in the degradation rate when the height of the lamps is adjusted. Under UVA and SLS lamps, an energy emission reduction of 80% barely changes the degradation rate. In the case of BVL, rising energy emission of up to 60% improves slightly in the degradation rate, probably due to the UV-A irradiation increment. However, under CVL, there are no differences in the degradation rate due to the energy emission rising by only 20%, and there is no additional contribution of UV-A irradiation.

On the contrary, changes in the height of lamps significantly impact the photolysis process, as shown in Figure 9b. Reducing 80% of energy emission to values of 18.1 W∙m^−2^ in the UVA lamp decreases the removal efficiency from 80% to 30%. Meanwhile, under the SLS lamp, the removal efficiency diminishes from 95% to 65% when the total energy emission is reduced from 1266.6 W∙m^−2^ to 291.7 W∙m^−2^ of the total irradiation. Although at a lamp distance of 60 cm to the reactor, the UV-A irradiation is 15.2 W∙m^−2^, which is similar to the UVA lamp, a higher CIP degradation is observed under SLS conditions, which is probably due to the contribution of some visible light range wavelengths that could photolyze the pollutant. Under the BVL lamp, there is photolytic degradation of CIP; however, the changes in the lamp height do not affect the degradation rate. On the other hand, using the CVL lamp does not remove CIP at any of the irradiation intensities applied. Based on these results, it can be deduced that the photocatalyst is still effective in CIP removal, even with energy emission values close to the natural solar light.

## 4. Conclusions

Based on the achieved results, the following conclusions can be summarized:

- A successful reduction in GO to rGO was achieved under mild conditions and using non-hazardous chemicals (125 °C and 45 min in the microwave oven, using ascorbic acid as a reducing agent).
- The synthesis of N/TiO_2_/rGO photocatalysts was successfully achieved under mild conditions (200 °C, 10 min) by microwave-assisted synthesis, where the anatase phase was obtained without further calcination, which is usually required in conventional synthesis methods.
- The optimal rGO content in N/TiO_2_/rGO nanocomposite was 0.25 wt.%; an excess of rGO reduces the photocatalytic activity due to a shielding effect for light absorption and recombination center effect.
- For OMP removal (ciprofloxacin, diclofenac, and salicylic acid), a synergistic effect of adsorption and photocatalysis was observed, where the degradation rate is affected by the radiation source, irradiation intensity, and type of OMP.
- The photocatalytic mechanism is defined mainly by the type of pollutant, while the irradiation source has a minor, if any, effect on the mechanism.
- Photolytic processes are significantly influenced by the irradiation intensity, indicating that the photolysis will probably not occur under some irradiation values.
- Under similar values to natural global solar irradiation, the photocatalytic process is still efficient in the removal of evaluated pollutants.
- Although the introduction of rGO in specific amounts showed enhanced photocatalytic activity under solar irradiation compared to N/TiO_2_ material and efficient OMP removal under natural solar conditions, there are several areas that need to be addressed to improve the practical applications of nanocomposites photocatalysts, such as degree of rGO reduction, efficient rGO dispersion, photocatalyst immobilization and reactor design.

## Figures and Tables

**Table 1:** Specific surface area, pore volume, and pore size of P25, N/TiO_2_, N/TiO_2_/rGO photocatalysts, and rGO material.

Material	*S*_BET_, m^2^∙g^−1^	*V*pore, cm^3^∙g^−1^	Average Pore Diameter, nm
TiO_2_ P25	48	0.196	13.7
rGO	192	0.323	6.5
N/TiO_2_	139	0.297	8.0
N/TiO_2_/rGO 0.25 wt.%	176	0.309	6.7
N/TiO_2_/rGO 1 wt.%	171	0.303	6.7
N/TiO_2_/rGO 3 wt.%	170	0.297	6.7
N/TiO_2_/rGO 5 wt.%	176	0.303	6.6
N/TiO_2_/rGO 10 wt.%	177	0.297	6.4

**Table 2:** Elemental composition of N/TiO_2_ and N/TiO_2_/rGO photocatalysts determined by SEM-EDS analysis.

Material	Elemental Composition, wt.%			
	Ti	O	C	N
N/TiO_2_	72.0	21.0	–	7.0
N/TiO_2_/rGO 0.25 wt.%	83.7	15.3	1.0	–
N/TiO_2_/rGO 1 wt.%	83.1	15.2	1.7	–
N/TiO_2_/rGO 3 wt.%	85.0	12.3	2.7	–
N/TiO_2_/rGO 5 wt.%	85.1	12.2	2.7	–
N/TiO_2_/rGO 10 wt.%	86.3	11.9	1.8	–

**Table 3:** Bulk composition of N/TiO_2_ and N/TiO_2_/rGO photocatalysts determined by XPS analysis.

Material	Elemental Composition, wt.%			
	Ti	O	C	N
N/TiO_2_	21.5	66.5	11.5	0.5
N/TiO_2_/rGO 0.25 wt.%	23.2	56.8	18.9	1.1

**Table 7:** UV and global irradiation values of each lamp at two distances to the reactor.

**Lamp_Distance**	**Same Distance to the Reactor**		
	**UV-A Irradiation (W∙m^−2^)**	**Total Irradiation (W∙m^−2^)**	**UV-A/T Ratio (%)**
UVA_20 cm	98.5	118.6	83.05
SLS_20 cm	59.2	1266.6	4.67
CVL_20 cm	0	241.1	0
BVL_20 cm	3.6	176.6	2.04
**Lamp_Distance**	**Same Global Irradiation**		
	**UV-A Irradiation (W∙m^−2^)**	**Total Irradiation (W∙m^−2^)**	**UV-A/T Ratio (%)**
UVA_50 cm	18.1	20.4	88.73
SLS_60 cm	15.2	291.7	5.21
CVL_15 cm	0	288.9	0
BVL_15 cm	5.8	289.1	2.01

## Data Availability

The data presented in this study are available upon reasonable request from the corresponding author.

## Supplementary Materials

The following are available online at https://www.mdpi.com/article/10.3390/nano12223975/s1, Figure S1: Inner pressure, temperature, and power supplied by the Microwave oven during the synthesis of nanocomposite N/TiO_2_/rGO with 0.25 wt.% of rGO. Figure S2: Global and UV-A radiation measurement of (**a**) UV-A lamp, (**b**) solar light simulator (SLS) lamp, (**c**) cold visible light (CVL) lamp, and (**d**) blue visible light (BVL) lamp. Figure S3: Radiation spectra of the different lamps: (**a**) UVA light (UVA), (**b**) Solar light (SLS), (**c**) Blue visible light (BVL), and (**d**) Cold visible light (CVL). Figure S4: Pore size distribution of N/TiO_2_, N/TiO_2_/rGO with different amounts of rGO (0.25, 1, 3, 5, and 10 wt. %), and P25 photocatalysts. Figure S5: Nitrogen adsorption–desorption isotherms of N/TiO_2_, N/TiO_2_/rGO with different amounts of rGO (0.25, 1, 3, 5, and 10 wt.%), and P25 photocatalysts. Figure S6: UV/Vis absorbance spectra of the initial concentration of (a) ciprofloxacin (CIP), (b) diclofenac (DCF), and (c) salicylic acid (SA).
