# Supplementary material for: Rapid Microwave-Assisted Synthesis of N/TiO2/rGO Nanoparticles for the Photocatalytic Degradation of Pharmaceuticals
Source: Nanomaterials (Basel). 2022 Nov 11;12(22):3975. doi: 10.3390/nano12223975 (PMC9696933; doi:10.3390/nano12223975)
Supplement: Supplementary file 1 [file nanomaterials-12-03975-s001.zip › nanomaterials-2011251-supplementary.pdf]

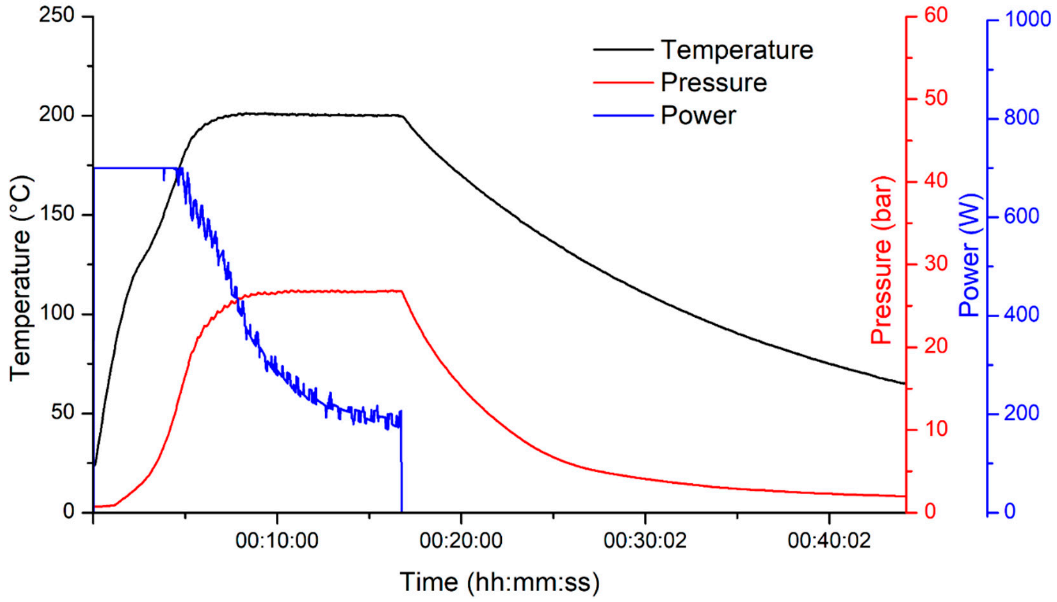

Figure S1. Inner pressure, temperature, and power supplied by the Microwave oven during the synthesis of nanocomposite N/TiO_2/rGO with 0.25 wt. % of rGO.

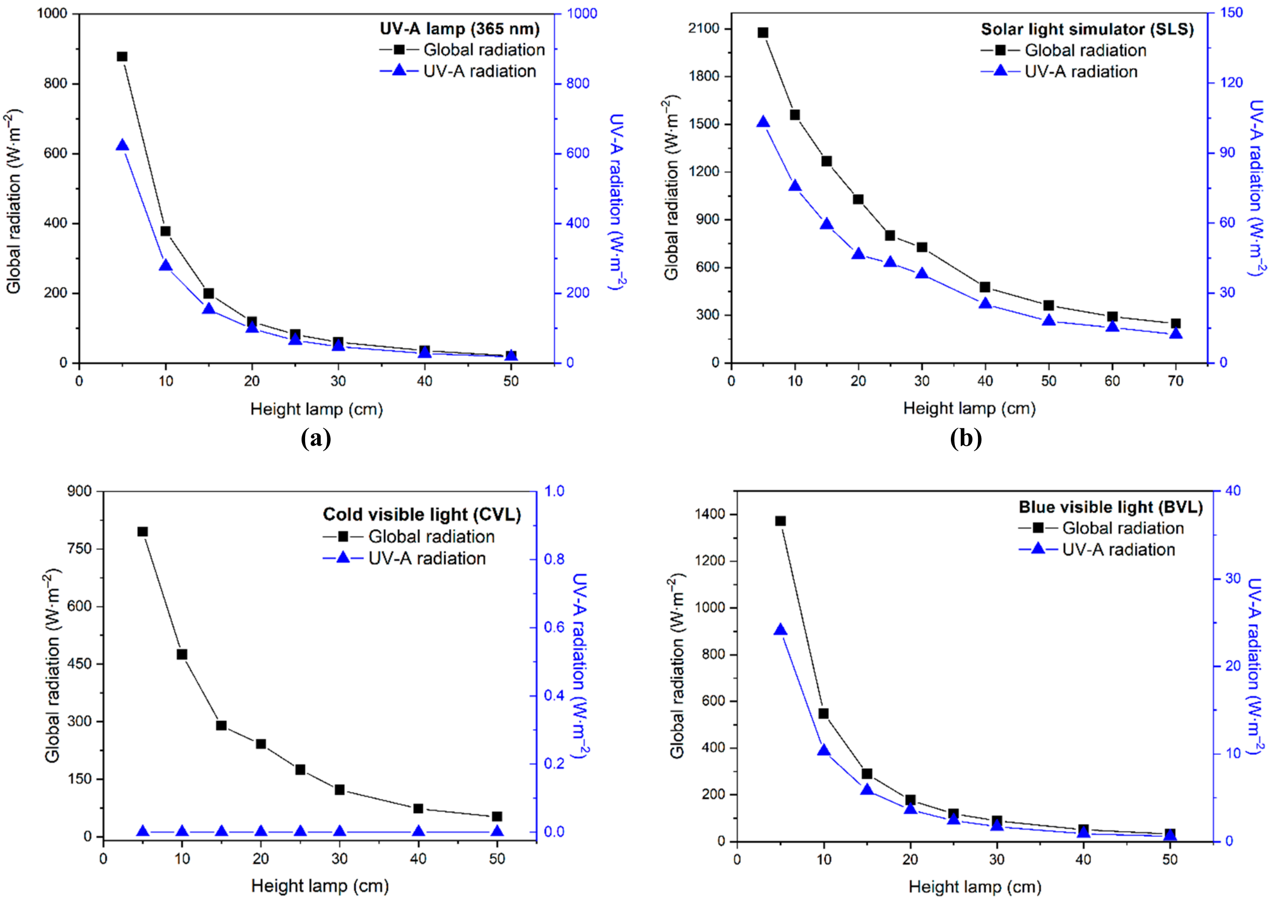

Figure S2. Global and UV-A radiation measurement of (**a**) UV-A lamp, (**b**) solar light simulator (SLS) lamp, (**c**) cold visible light (CVL) lamp, and (**d**) blue visible light (BVL) lamp.

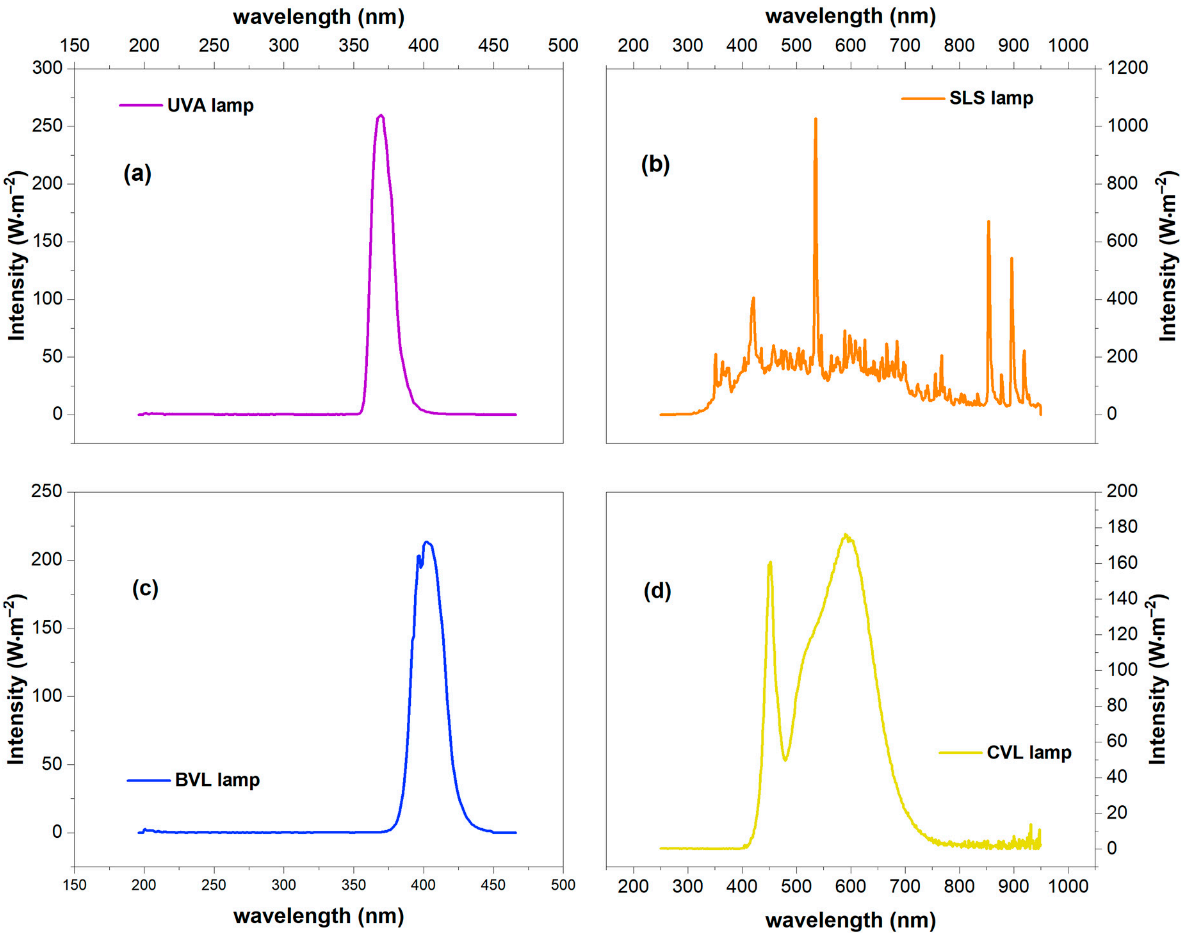

Figure S3. Radiation spectra of the different lamps at 20 cm height: (**a**) UVA light (UVA), (**b**) Solar light simulator (SLS), (**c**) Blue visible light (BVL), and (**d**) Cold visible light (CVL).

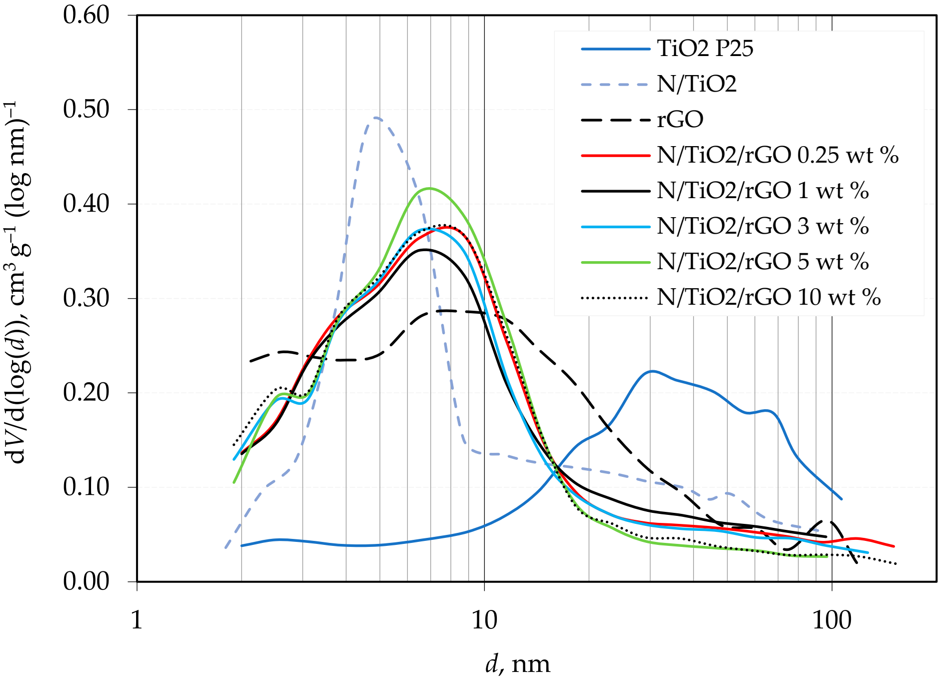

Figure S4. Pore size distribution of N/TiO_2, N/TiO_2/rGO with different amounts of rGO (0.25, 1, 3, 5, and 10 wt. %), and P25 photocatalysts.

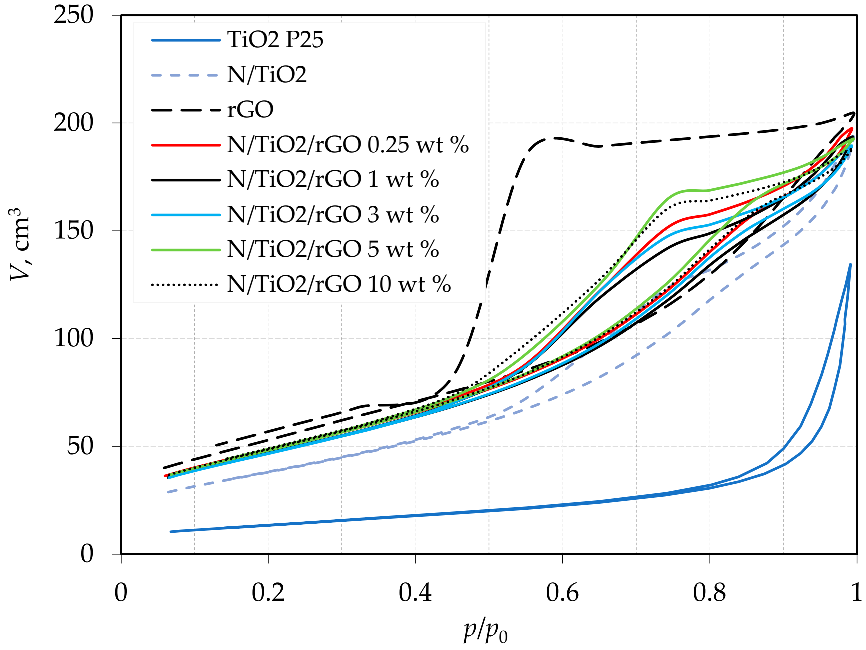

Figure S5. Nitrogen adsorption-desorption isotherms of N/TiO_2, N/TiO_2/rGO with different amounts of rGO (0.25, 1, 3, 5, and 10 wt. %), and P25 photocatalysts.

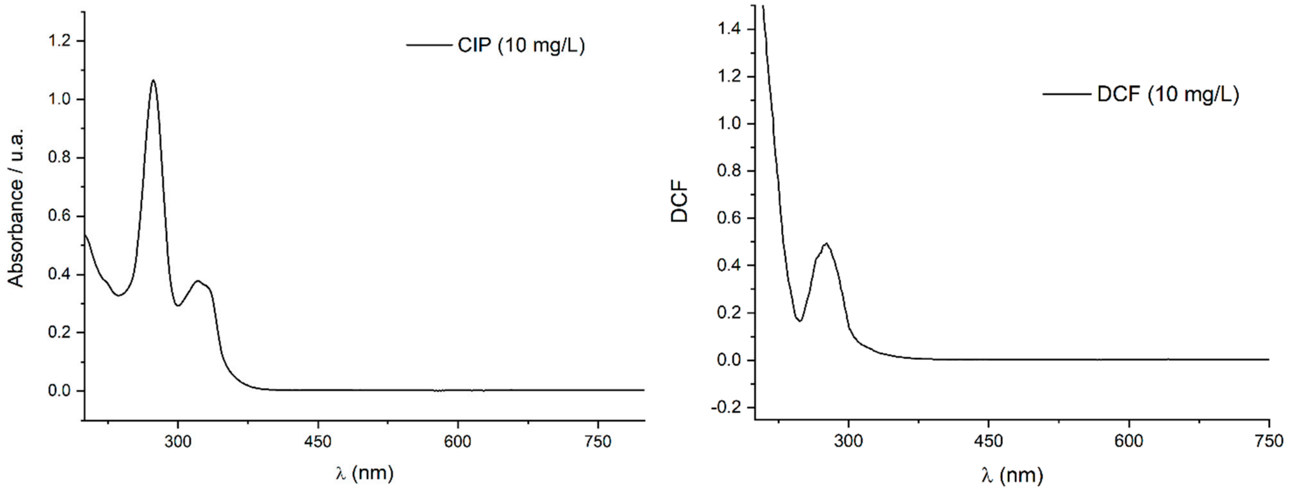

(**a**) (**b**)

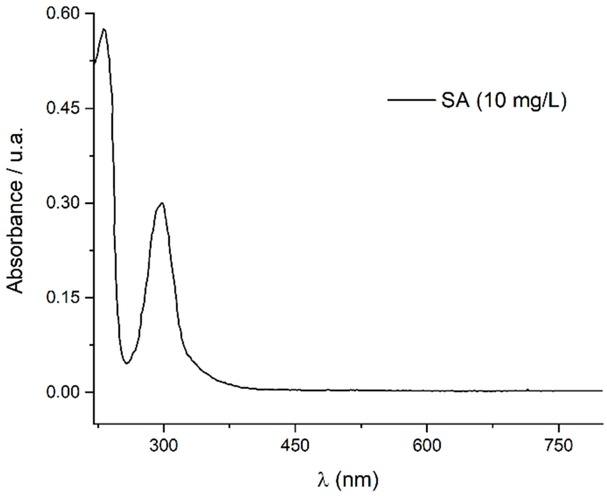

(**c**)

Figure S6. UV/Vis absorbance spectra of the initial concentration of (**a**) ciprofloxacin (CIP), (**b**) diclofenac (DCF), and (**c**) salicylic acid (SA).
